# Placental impression smears is a good indicator of placental malaria in sub-Saharan Africa

**DOI:** 10.11604/pamj.2019.34.30.20013

**Published:** 2019-09-16

**Authors:** Smaïla Ouédraogo, Manfred Accrombessi, Ismaël Diallo, Roussine Codo, Adama Ouattara, Laurent Ouédraogo, Achille Massougbodji, Michel Cot

**Affiliations:** 1 Unité de Formation et de Recherche en Science de la Santé, Université Joseph Ki-Zerbo, Ouagdougou, Burkina Faso; 2 Centre Hospitalier Universitaire Yalgadogo, Ouédraogo, Burkina Faso; 3 Fondation Scientifique pour la Recherche Scientifique, Cotonou, Benin; 4 Faculté de Médecine de Cotonou, Université d’Abomey-Calavi, Cotonou, Bénin; 5 Centre Hospitalier Universitaire de Bogodogo, Ouagadougou, Burkina Faso; 6 Université de Ouahigouya, Ouahigouya, Burkina Faso; 7 MERIT- Mère et Enfant Face aux Infections Tropicales, Institut de Recherche pour le Développement, Paris, France, Université Paris Descartes, Sorbonne Paris Cité, France

**Keywords:** Performance, placenta impression smears, placenta histology, placental malaria, sub-Saharan Africa

## Abstract

**Introduction:**

Placental malaria (PM) is an important predictor of infant morbidity and mortality in sub-Saharan Africa. Although placental histology is the gold standard test to diagnose PM, the placenta impression smears remains widely used in epidemiological studies. This study is set to evaluate the performance of placental impression smears to detect PM in pregnant women in southern Benin.

**Methods:**

A cross-sectional analysis was performed on data collected in the framework a multicenter randomized clinical trial (Malaria in Pregnancy Preventive and Alternative Drugs). Samples from 491 pregnant women were examined in the district of Allada, Southern Benin. *Plasmodium falciparum* infections have been assessed in placental blood and placental biopsy.

**Results:**

Placental malaria detected by placenta impression smears and histology were prevalent in 11.4% and 10.8%, respectively. Sensitivity and specificity of placental impression smears were 90.6% and 98.4%. Among 55 pregnant women tested positive by placenta impression smears, 48 were positive by the histology, while 7 were negative (positive predictive value: 87.3%). Four hundred and twenty four (424) of the 429 tested negative by the placenta impression smears, were also negative according to histology whereas the rest (5 of 429) of the women were positive (negative predictive value: 98.8%).

**Conclusion:**

Placenta impression smear is an accurate and easy method for the diagnosis of placental malaria.

## Introduction

Malaria due to *P. falciparum* infection during pregnancy is a serious public health problem in sub-Saharan Africa (SSA). A quarter of women has evidence of placental malaria (PM) at the time of delivery [1,2]. PM contributes to maternal morbidity, preterm birth and low birthweight [3,4]. It's also associated with high susceptibility of infant to malaria and non-malaria infections during the first years of life [5-8]. During pregnancy, adhesion of *P. falciparum*-infected erythrocytes to syncytiotrophoblast leads to parasite sequestration in the intervillous space. The parasite adheres specifically to chondroitin sulfate A expressed on syncytiotrophoblast [9]. Then, PM may be detected in the absence of peripheral blood parasitemia [10]. PM is widely recognized as an indicator for malaria infection in epidemiologic surveys for both operational and research purposes [11]. Malaria during pregnancy raises a greater diagnostic challenge especially in SSA [12]. Placental histology is the “gold standard ” for the diagnosis of malaria during pregnancy, but it is relatively expensive and labor intensive and is not often available. Because it is easy to perform, placenta impression smears stay the method frequently used to diagnose PM. However, there are few data focused on the accuracy of the diagnosis of placenta impression smear as method to detect PM. On the occasion of a multi-center trial of Intermittent Preventive Treatment in Pregnancy (IPTp) comparing sulfadoxine-pyrimethamine (SP) and mefloquine (MQ) ("Malaria in Pregnancy Preventive Alternative Drugs" (MiPPAD) [13], we had the opportunity to investigate the performance of placenta impression smears to identify pregnant women with PM in the context of IPTp and the use of long-lasting insecticide treated nets (LLITNs) in Beninese pregnant women.

## Methods

**Study design:** a cross sectional analysis of data collected on four hundred and ninety-one pregnant women between January 2010 and May 2012 has been performed. Pregnant women were followed-up from the first antenatal clinical (ANC1) visit until the time of delivery.

**Study site, population and procedures:** the study site, population and procedure have been described elsewhere [14,15]. Briefly, the study was conducted in three maternity clinics (Allada, Attogon and Sékou) in the district of Allada, a semi-rural area located 50 km north of Cotonou, the economic capital of Benin. Allada district is characterized by a subtropical climate and malaria is hyperendemic with an average of 20.5 infected anopheles/person/year.

**Plasmodium falciparum:** is the predominant species transmitted (97%). The study population was composed of HIV-negative pregnant women residing in the district of Allada. During the study, socio-demographic (age, parity, marital status, education level), clinical (gestational age, weight, height) and biological (blood smear, haemoglobin level) data were collected. Two doses of IPTp (1500/75 mg SP per dose or 15 mg/kg MQ per dose) were administered throughout ANC visits. The second dose of IPTp was given at least of 1 month apart from the administration of the first dose. At enrolment, each woman received a LLITN and their use was assessed at each ANC visit. Clinical malaria episodes were treated with oral quinine or artemether-lumefantrine in the first and subsequent trimesters, respectively, for uncomplicated malaria, and with parental quinine for severe malaria. At delivery, women's peripheral blood, cord blood, and placenta (biopsy and impression smears) samples were collected for hematological and parasitological evaluation.

**Laboratory methods:** thick and thin blood films were stained and read for *Plasmodium* species detection according to standard quality-control procedures [16]. Tissue samples were collected from the maternal side of the placenta and placed into 10% neutral buffered formalin. Biopsies were processed, stained, and examined following standard procedures [17]. Impression smears from the placenta blood were stained with Giemsa and read following a standardized protocol [18,19].

**Placental impression smears:** a 2.5 x 2.5 cm^3^ sample from the selected placenta area was cut. The sample included the thickness of tissue from the maternal to the fetal side limited by the fetal membranes. One of the internal faces of sample was carefully put into contact with the slide, on the closest location to the identification area of slide. Then, the placental section was dry with a small piece of filter paper. One of the faces of the dried placental section was put into contact with the slide, on the most distal location to the identification area in the slide. The same procedure was repeated on a second slide.

**Figure 1:**
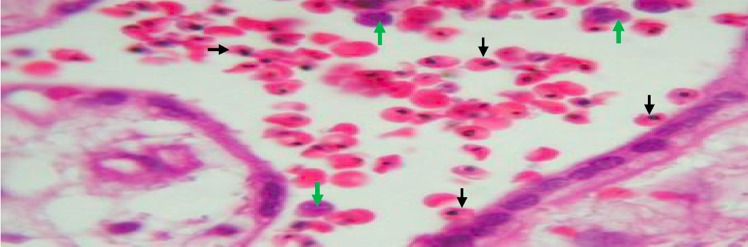
Placental tissue with active malaria infection â€ (by histology)

**Figure 2:**
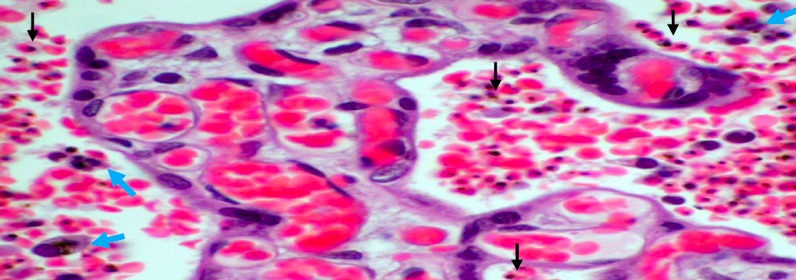
Placental tissue with chronic malaria infection â€ (by histology)

**Figure 3:**
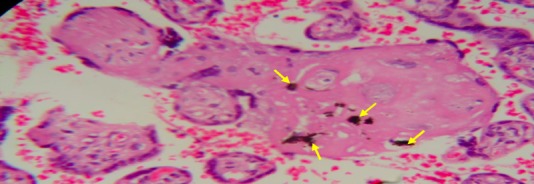
Placental tissue with past malaria infection â€ (By histology)

**Placental histology:** the 2.5 x 2.5 cm^3^ sample collected for placental impression smears was immediately put in a 50 ml of 10% buffered formalin container. This container was stored in a 4°C fridge until the placental tissue is processed at the department of pathology of the faculty of Medicine of the University of Abomey Calavi. The maximum of fixation was of 5 days. PM was characterized using the classification of Bulmer *et al*. [20]: uninfected (no parasites or pigment), acute (Figure 1, parasites in intervillous spaces), chronic (Figure 2, parasites in maternal erythrocytes and pigment in fibrin or cells within fibrin and/or chorionic villous syncytiotrophoblast or stroma), past (Figure 3, no parasites and pigment confined to fibrin or cells within fibrin). In this analysis, the chronic and active malaria infections have been taking into account to compared placental impression smears and histology. Each placenta impression smear was independently examined by two technicians. In case of discordances, a third reading was required. Placental histology was examined without knowledge of the placental impression and peripheral blood smears results. In addition, an external quality control was made on 100% of positive slide and 10% of negative slide in reference laboratory at Barcelona Centre for International Health Research (CRESIB), Hospital Clínic Universitat de Barcelona.

**Ethical considerations:** this study was approved by the Ethics Committee of the Faculty of Medicine of Cotonou in Benin. Before each inclusion, all participants involved in the study provided their written informed consent. In case the woman could not read, an impartial witness was involved in the process.

**Statistical analysis:** data were double-entered into Microsoft Access 2003 database and analyzed with Stata 12.0 Software for Windows. Sensitivity (Se), specificity (Sp), positive predictive value (PPV), negative predictive value (NPV), positive and negative likelihood ratio (LH ±) were calculated to determine the accuracy diagnosis of placental impression smears vs. placental histology. Sensibility was calculated as true positives / (true positives + false negatives), Sp as true negatives / (true negatives + false positives), PPV as true positives / (true positives + false positives), NPV as true negatives / (true negatives + false negatives) [21].

## Results

**Figure 4:**
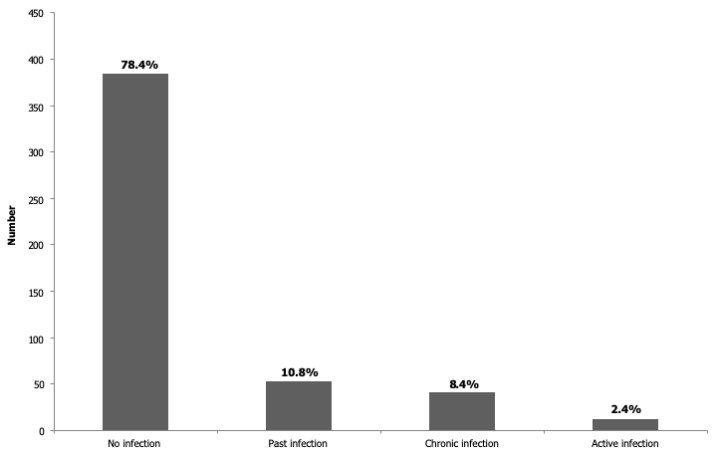
Placental malaria infection according to the histology assessment in Beninese pregnant women (Bulmer classification) e

Four hundred and eighty-four placentae have been examined. Women’s mean age was 25.9 years (95% CI: (25.4-26.4). Ninety-one (18.5%) of the women were primigravidae. The mean body mass index (BMI) was 24.4 kg/m² (95% CI: (24.1-24.7). Gestational age at delivery was 39.5 weeks (95% CI: (39.1-39.8) and 7.3% of deliveries were preterm births (Table 1). Table 2 shows the biological characteristics of pregnant women at delivery. Their mean haemoglobin at delivery was 11.2 g/dL (95% CI: (11.0-11.3)) and 39.5% of women presented anaemia. The proportion of peripheral malaria infection was 10.2%. The prevalence of PM by placenta impression smears and placenta histology were 11.4% and 10.8%, respectively. When we considered past, chronic and active infections for histology, malaria infection was prevalent in 21.6% (106/491). The median of placental infection density detected by impression smear was 12880 parasites/μl. According to Bumler classification, 78.4% of women were uninfected, while 10.8%, 8.4% and 2.4% of them presented past, chronic and acute infection, respectively (Figure 4). Among the 53 tested positive by histopathology, 48 have been found to be positive by placenta impression smear while 5 were negative (Table 3). Thus the sensitivity of placental impression smears was 90.6% (95% CI: 82.7% - 98.5%). Out of 431 which were negative by histology, 7 were positive by placental impression smears, represented a specificity of 98.4% (95% CI: 97.2% - 99.6%) (Table 4). The positive and negative predictive value were 87.3% (95% CI: 78.5% - 96.1%) and 98.8% (95% CI: 97.8% - 99.8%), respectively (Table 4). Area under the ROC curve (UAC) was 0.94 (95% CI: (0.90 - 0.98)) and translated a good discrimination of placental impression smear to detect PM.

**Table 1:** Clinical and demographical characteristics of pregnant women at delivery in Southern Benin

Characteristics		No.	Mean or %
Age (years)	Mean	491	25.9 [25.4-26.4]
	< 20	47	9.6
	20-30	303	61.7
	≥ 30	141	28.7
Gestational age (weeks)	Mean	464	39.5 [39.1-39.8]
	≥ 37	430	92.7
	< 37	34	7.3
BMI (kg/m²)	Mean	454	24.4 [24.1-24.7]
	≥ 20	435	95.8
	< 20	19	4.2
Gravidity (%)	Primigravidae	91	18.5
	Secundigravidae	92	18.8
	Multigravidae	308	62.7
Ethnic group (%)	Aïzo	305	62.1
	Fon	126	25.7
	Others	60	12.2
Residence area (%)	Sekou	320	65.1
	Attogon	101	20.6
	Allada	70	14.3
Marital situation (%)	Maried	482	98.2
	Single	9	1.8
Education (%)	No	328	66.8
	Yes	163	33.2
IPTp group* (%)	SP	173	35.2
	MQFD	169	34.4
	MQSD	149	30.4

BMI: Body Mass Index (weight in kilograms divided by the square of the height in meters (kg/m²).

* IPTp: Intermittent Preventive Treatment in Pregnancy (allocation group of MiPPAD trial)

SP: Sulfadoxine Pyrimethamine, MQFD: Mefloquine full dose, MQSD: Mefloquine split dose

95% Confidence Interval are in parentheses

**Table 2:** Parasitological and hematological characteristics of pregnant women at delivery in Southern Benin, N=491

Characteristics		No.	Mean or %
Blood smear in peripheral blood (%)	Negative	432	89.8
	Positive	49	10.2
Placenta impression smears (%)	Negative	429	88.6
	Positive	55	11.4
Placental histology † (%)	Negative	438	89.2
	Positive	53	10.8
Placental histology ‡ (%)	Negative	385	78.4
	Positive	106	21.6
Haemoglobin level (g/dL)	Mean	481	11.2 [11.0-11.3]
Anaemia* (%)	No	291	60.5
	Yes	190	39.5
Inflammatory syndrome (%)	No	326	67.2
	Yes	159	32.8

† Proportion taking account into chronic and active malaria infection

‡ Proportion taking account into past, chronic and active malaria infection

* Anaemia: haemoglobin level less than 11 g/dL

95% Confidence Interval are in parentheses

**Table 3:** Placental impression smears results compared to placental histology

		Placental histology		Total
		Positive, n(%)	Negative, n(%)	
**Placental impression smears**	Positive	48 (90.6)	7 (1.6)	55 (11.4)
	Negative	5 (9.4)	424 (98.4)	429 (88.6)
Total		53 (100)	431 (100)	484 (100)

**Table 4:** Diagnostic performance of placental impression smears using placental histology as gold standard

Performance	Value (95% CI)
Sensitivity (%)	90.6 (82.7-98.5)
Specificity (%)	98.4 (97.2-99.6)
Positive predictive value (%)	87.3 (78.5-96.1)
Negative predictive value (%)	98.8 (97.8-99.8)

95% CI: Confidence interval at 95%

## Discussion

To appreciate the accuracy of placental impression smears to diagnose PM in pregnant women, we compared placental impression smears to placental histology. The prevalence of PM was 11.4% and 10.8% by impression smears and placental histology, respectively. Less than 12% of pregnant women presented a PM. This finding supports similar results reported in a neighborhood area (Nigeria, 11.5%) and in Kenya (11.4%) [22,23]. This low prevalence may partially be explained by the effect of IPTp, the use of LLITNs and appropriate active and passive follow-up of women throughout their participation in MiPPAD clinical trial. Many studies have previously showed the efficacy of IPTp and LLITNs on the reduction of malaria in pregnancy [13,24-26]. In the study, the diagnosis of past infections by placental impression smears was not preformed. On the one hand, placental impression smears did not allow to identify the sediment of malarial pigment in placental structure (fibrin sediment and trophoblast cells only recognizable by histology) [20] and probably underestimated the past malaria infection. On the other hand, the MiPPAD protocol had not planned to do it. Elsewhere in Africa, studies have showed that the ability of placental impression smears to correctly diagnose past infections remains lower than placental histology [19,27]. Sensitivity and specificity of the placental impression smear were 90.6% and 98.4%, respectively compared with placental histology. This means that among women with placental malaria, approximately 91% were properly diagnosed by placental impression smear and those with uninfected placenta, ~98% were also correctly diagnosed. These results showed that placental impression smear is a good diagnostic tool of PM in Beninese pregnant women. Histological examination of placenta biopsy is the gold standard for the diagnosis of PM. The placenta histology indicates the presence of malaria parasites and pigment in the placental tissue. Malaria infection can then be classified in active, chronic, past and no infection. However, due to limited resources and technical expertise, placental histology is rarely available in malaria endemic areas where recourses are rare [12]. Placental impression smear is one of the most sensitive tools for malaria parasites detection and does not require any specific equipment. Likewise, it is a method usually used by several studies on PM researches [27] but there are few studies which appreciated its performance.

In this study, the sensitivity of placental impression smear was 90.6%. This performance is much higher than that reported in the literature. Indeed, Rogerson *et al* in Malawi and Anchang-Kimbi *et al*. in Cameroun reported prevalence rates of 64.6% and 50%, respectively. However, the specificity of 98% that we report is comparable to those reported by Rogerson and Anchang-Kimbi in their respective studies [18,28]. Some biopsies have not been systematically put on formalin; others were kept for a long time before their examination. These incidents certainly affected the sensitivity of placental histology, which may explain, in part, the difference that we observe between our results and those of Malawi and Cameroun. Sensibility and specificity are probably the main parameters which better described the accuracy of a diagnostic test [29], but in practice, the question of interest is to know the probability to have or not PM when placental impression smear result is positive or negative [30]. This information is provided by predictive values [31]. Positive and negative predictive values of placental impression smear in the study were 87.3% and 98.8%, respectively. That reflects the accuracy of placental impression smear to detect placental malaria infection. Similar predictive values have been found in Malawi (93.6% and 89.6% for positive and negative predictive values, respectively) when placental impression smear was used to detect PM [19]. The placental impression smear had an AUC of 0.94. Several authors have previously described that AUC allow assessing the interest of diagnostic test and a test with an AUC between 0.9 and 1 were very informative [32,33]. Hence, placental impression smear is globally a better diagnostic test to discriminate pregnant women with and without PM in Southern Benin.

## Conclusion

PM was relatively common in the study, probably due to the effect of preventives strategies against malaria in pregnancy and appropriate curative treatment during their follow-up. Placental impression smear seems to be a good diagnostic tool to detect the placental malaria infection because of its good sensitivity, specificity, positive and negative predictive values compared to the placental histology. Placental impression smear is easy to perform and less expensive compared to placental histology. Placental impression smear could be considered as a good diagnostic tool to detect PM in low income countries where malaria is endemic. The use of impression smear for the diagnosis of PM in epidemiological studies will allow more characterizing the burden of morbidity and mortality attributable to malaria during pregnancy and defining adequate malaria in pregnancy preventive strategies.

### What is known about this topic

- Malaria during pregnancy raises a greater diagnostic challenge especially in sub-Saharan Africa;
- Placental histology is the “gold standard ” for the diagnosis of malaria during pregnancy;
- Placental histology is relatively expensive, labor intensive and often unavailable.

### What this study adds

- Placenta impression smear is an accurate and easy method for the diagnosis of placental malaria.

## Competing interests

The authors declare no competing interests.
